# Advance statements in mental healthcare: time to close the evidence to practice gap

**DOI:** 10.1017/S2045796023000835

**Published:** 2023-12-06

**Authors:** Antonio Lasalvia, Sara Patuzzo, Esther Braun, Claire Henderson

**Affiliations:** 1 Section of Psychiatry, Department of Neurosciences, Biomedicine and Movement Sciences, University of Verona, Verona, Italy; 2 Department of Surgery, Dentistry, Paediatrics and Gynaecology, University of Verona, Verona, Italy; 3 Institute for Medical Ethics and History of Medicine, Ruhr University Bochum, Bochum, Germany; 4 Department of Philosophy, University of Oxford, Oxford, UK; 5 Health Service and Population Research Department P029, David Goldberg Centre, King’s College London Institute of Psychiatry, London, UK

**Keywords:** community mental health, discrimination, ethics, rights of persons with disabilities, social and political issues

## Abstract

This article discusses advance statements in mental health care, which allow individuals with mental disorders to express their preferences for treatment during mental health crises. Despite the evidence supporting their effectiveness, their implementation in clinical practice remains limited. This article explores variations among advance statements, such as psychiatric advance directives (PADs), joint crisis plans (JCPs) and self-binding directives (SBDs), highlighting their content, development process and legal status. We outline the benefits of advance statements, including empowerment, early intervention, improved therapeutic relationships and reduced compulsory admissions. We then draw attention to the challenges that may contribute to their lack of implementation, including legal complexities, communication issues, cultural factors, potential inequities, healthcare provider knowledge, changing preferences, resource constraints, crisis responses, data privacy, family involvement, and long-term evaluation. In conclusion, advance statements offer significant benefits but require addressing these critical aspects to ensure ethical and effective use. Bridging the evidence-to-practice gap is essential, with a focus on implementation science. Integrating these tools into routine clinical practice can significantly benefit individuals with severe mental disorders and mental health systems.

## Introduction

Advance statements in mental healthcare are documents that allow adults with mental disorders to state their will and preferences for situations in which mental health crises impair their decision-making capacity. This may include advance consent or dissent to medical treatments or hospital admissions (Gaillard *et al.*, 2023). Despite being available in many jurisdictions and considerable trial evidence for their effectiveness in relation to several important outcomes, the uptake of advance statements remains low – as does knowledge about them among many frontline mental health professionals. In this article, we first summarize the benefits and opportunities presented by advance statements, including their potential to counteract the stigma and discrimination associated with mental illnesses. We then draw attention to the challenges that contribute to their lack of implementation, to show how addressing these challenges must now become the focus of research efforts.

## Key dimensions of variation among advance statements

While all advance statements share the overarching goal of empowering individuals with mental health conditions to have a say in their care, key differences lie in their content, development process and legal status. The terminology and legal recognition of these documents can vary significantly between jurisdictions. The specific characteristics are briefly outlined below.

### Psychiatric advance directives (PADs)

- Purpose: PADs allow individuals with mental health conditions to specify their preferences and instructions for treatment in advance, which come into effect during periods when they do not have decision-making capacity.
- Content: They can include preferences for medications, therapy, hospitalization or even the choice of a specific mental healthcare provider.
- Legal status: PADs have legal standing in many jurisdictions, allowing individuals to have some control over their treatment even when they are unable to make autonomous decisions.

### Joint crisis plans (JCPs)

- Purpose: JCPs are collaborative documents developed between individuals with mental health conditions and their mental healthcare providers or support networks, facilitated by a mental health professional independent of the treating team.
- Content: These plans typically outline crisis management strategies, including warning signs, preferred treatments and contacts for family or friends who should be involved during a crisis.
- Legal status: While they may not always have legal standing, JCPs are important tools for communication and coordination in mental healthcare.

### Self-binding directives (SBDs)

- Purpose: SBDs are also collaborative documents where individuals outline their treatment preferences and explicitly state the conditions under which treatment may be administered or withheld, even against their current wishes.
- Content: These directives often contain specific instructions about the circumstances under which involuntary treatment can or cannot be applied.
- Legal status: SBDs vary in legal status depending on local laws. In some jurisdictions, they may have strong legal weight, while in others, their enforceability may be limited.

While PADs can be drawn up by users individually, SBDs (also known as ‘Ulysses contracts’ or ‘Ulysses arrangements’) and JCPs are created collaboratively by the user and a member of the treatment team and, if possible, a relative or informal caregiver (Maître *et al.*, 2013). In the case of JCPs, their creation is facilitated by a mental health professional independent of the treating team. (Sutherby *et al.*, 1999). However, SBDs differ from both PADs and JCPs in two ways: first, they allow users to give advance consent for involuntary hospital admission or treatment in the event of a future mental health crisis, and second, they cannot be revoked under the circumstances in which they are to be applied (Potthoff *et al.*, 2022). JCPs and SBDs promote user involvement and dialogue (Murray and Wortzel, 2019). The benefits of PADs with respect to autonomy have now been demonstrated empirically, and initial studies have shown that PADs improve user involvement, empowerment, recovery and the therapeutic alliance and integration of care (Nicaise *et al.*, 2013). Recently, a randomized controlled trial in France showed that PADs facilitated by peer workers were associated with fewer symptoms, higher empowerment and higher recovery rates than those of the control group (Tinland *et al.*, 2022).

## Benefits and opportunities

The literature consistently reports that various forms of psychiatric advance statements offer numerous benefits and opportunities to people with severe mental disorders, healthcare providers and the mental health system. These benefits arise from increased patient empowerment, early intervention, improvement of the therapeutic relationship and reduced compulsory admission rates. We now explore these advantages in more detail.

### Empowerment and autonomy

These instruments empower people with mental disorders to participate actively in their treatment decisions. By expressing their preferences in advance, patients gain a sense of control over their care, ensuring that their treatment aligns with their values, beliefs and personal goals (Braun *et al.*, 2023). PADs and JCPs ensure the moral and legal rights to make autonomous decisions with respect to one’s medical treatment. In addition, these tools are useful for respecting the individual’s concept of quality of life and well-being, which may not align with the ‘clinical good’ as determined by physicians. Furthermore, creating advance statements may increase patients’ sense of ownership and responsibility for their mental health, potentially leading to better treatment adherence and engagement in recovery. This sense of empowerment fosters self-determination and challenges the notion that people with mental disorders are passive care recipients.

### Early intervention

Advance statements such as JCPs are specifically designed to facilitate early intervention during a relapse. By outlining warning signs and preferred treatment approaches, they enable healthcare providers to respond swiftly to emerging crises. This early intervention can significantly reduce the severity and duration of mental health crises, improve outcomes and reduce the need for involuntary hospitalization.

### Improved therapeutic relationship

The collaborative process of developing advance statements enhances the therapeutic relationship between mental health service users and healthcare professionals (Swanson *et al.*, 2006; Thornicroft *et al.*, 2013). In contrast to traditional top-down approaches, the development of these documents requires active engagement and shared decision-making. This fosters trust, mutual respect and open communication between patients and providers, creating a more patient-centred and empowering treatment environment. The collaborative process of developing advance statements involves open communication between patients and healthcare providers. Through this dialogue, healthcare professionals can gain a deeper understanding of patients’ experiences, needs and treatment preferences. This makes providers more likely to treat patients with respect, empathy and compassion, and may challenge misconceptions and stereotypes surrounding mental disorders.

### Reduced compulsory admissions

Shared decision-making interventions, including advance statements, JCPs and patient-held information strategies, are among the most effective interventions for reducing coercive treatment and compulsory admissions in people with severe mental disorders (Barbui *et al.*, 2021; Bone *et al.*, 2019; de Jong Mh *et al.*, 2016; Henderson *et al.*, 2004; Tinland *et al.*, 2022). Crisis-planning interventions (e.g., advance statements strategies and JCPs) were found to reduce compulsory admissions by 25% compared with usual care (Molyneaux *et al.*, 2019). This reduction in involuntary admissions not only respects patients’ autonomy but also reduces emotional distress and potential trauma associated with forced hospitalization.

### Integration of care

Advance statements contain crucial information about an individual’s treatment preferences, medication and care instructions. This information allows healthcare providers in different settings to provide consistent and personalized care, even during emergencies or transitions between services, leading to increased continuity of care.

### Facilitation by peer workers

The involvement of peer workers in facilitating SBDs and JCPs has yielded promising results (Easter *et al.*, 2017; Ruchlewska *et al.*, 2009). Peers with personal experiences of mental illness and recovery can provide unique insights, empathy and understanding of the developmental process. Peer support contributes to a more trusting and compassionate environment, which positively affects the mental health outcomes of service users (Loubière *et al.*, 2023; Tinland *et al.*, 2022).

### Role of advocacy and support organizations

Advocacy and support organizations for people with mental health conditions can play a vital role in promoting the use of advance statements. Organizations can provide information, resources and assistance in creating and updating these documents, empowering individuals to take charge of their mental healthcare.

### Training and education

Healthcare professionals, mental health service users and family members may benefit from specialized training and education for JCPs and SBDs. Training programs can enhance understanding, communication and implementation of these documents, leading to improved patient outcomes.

### Tackling mental illness stigma

The implementation of advance statements may represent a unique opportunity to tackle stigma associated with mental illness. More specifically, these measures hold great potential for addressing self-stigma by empowering individuals to take control of their mental healthcare and challenging negative self-perceptions. Self-stigma is a prevalent challenge faced by people with severe mental disorders (Fernández *et al.*, 2023). This occurs when people internalize negative beliefs and stereotypes about mental illness, leading to feelings of shame, worthlessness and reluctance to seek help (Corrigan *et al.*, 2016). Advance statements can help people with severe mental disorders overcome self-stigma and foster a positive path towards recovery. The collaborative nature of developing advance statements encourages self-advocacy among individuals with severe mental disorders. In these discussions, patients articulated their preferences, needs and desired treatment approaches, empowering them to communicate their wishes. In developing advance statements, people with mental disorders can outline coping strategies that they find effective during a mental health crisis. By identifying these coping mechanisms in advance, patients can strengthen their resilience and ability to effectively manage challenges. This proactive approach reduces feelings of helplessness and reinforces their belief in their capacity to overcome difficulties, countering the impact of self-stigma on their sense of self-worth and capability. By participating in the development of advance statements, people with mental disorders acknowledge the importance of seeking support during mental health crises. This normalization of help-seeking behaviour challenges the self-stigma that often discourages individuals from accessing care. Emphasizing the value of support and early intervention promotes a positive mindset towards seeking help when needed, breaking down self-stigmatizing barriers to seeking mental health services.

## Potential problems and challenges

Although advance statements offer significant benefits, their implementation can encounter several potential problems and challenges. These issues arise from legal, ethical, cultural, communication- and resource-related factors that can affect the effectiveness and acceptance of these documents (Shields *et al*., 2014). We briefly discuss these issues below.

### Legal and ethical complexities

Implementing advance statements raises legal and ethical questions regarding patient autonomy, decision-making capacity and the balance between individual rights and public safety. Regarding the person’s autonomy, there may be doubts regarding whether a patient had decision-making capacity at the time of completing the advance statement. However, advance statements should not automatically be regarded as invalid if there is no substantial evidence that a person lacks decision-making capacity at the time of their completion. Moreover, when a trusted person is designated by the patient, they can offer significant support (Allen, 2020) in two roles: as a surrogate decision-maker or as a trustee. In fact, if the patient, envisioning a future where they may lose decision-making capacity, has completed advance directives by naming an individual to act on their behalf as a ‘surrogate decision-maker’, this designated person becomes vital if that scenario unfolds, as they possess the authority to provide or withhold consent for the patient’s treatment. Similarly, if the patient has identified someone in their advance directives to serve as a channel for their wishes, acting as a ‘trustee’, this individual becomes a valuable intermediary with medical professionals, assuming the role of a ‘moral guardian’ to ensure that the patient’s previously expressed will is acknowledged and honoured (Allen, 2020). Regarding the balance between individual rights and public safety, determining the validity of these documents during crises can be complex, because adherence to patient directives may conflict with immediate safety concerns. This opens a debate on compulsory medical treatments when they are implemented against a person’s will to safeguard public safety. Complex ethical and legal questions arise when patients with mental illness pose a danger to others but refuse psychiatric treatment in an advance statement.

### Communication and understanding

The successful implementation of JCPs and SBDs relies heavily on effective communication among patients, healthcare providers, and possibly family members. Misinterpretation or miscommunication can lead to discrepancies between patients’ actual preferences and the preferences recorded in these documents. Ensuring clear and open communication is crucial for accurately representing patient intentions in JCPs and SBDs.

### Cultural and social factors

These factors may influence the acceptance and effectiveness of advance statements in routine settings. Different communities may have varying beliefs and attitudes towards mental health treatment and decision making, impacting the development and adherence to these documents. Culturally competent approaches are necessary to address these variations and to ensure that these instruments are tailored to the unique needs and preferences of each individual.

### Widening versus reducing inequities in mental healthcare

People who experience higher rates of compulsory admission stand to benefit relatively more from advance statements, but only if they are implemented in a way that is codesigned with them (Babatunde *et al.*, 2023). In England, the detention rates of Black people, defined as people of Black African and Caribbean heritage, including those of mixed ethnicity, are disproportionately higher than those of white British people and they have poorer care experiences and outcomes (Barnett *et al.*, 2019; Care Quality Commission, 2018). Black people of Caribbean heritage are more likely to be re-admitted or repeatedly detained than white people (Barnett *et al.*, 2019) and are less likely to be referred for specialist mental healthcare (Memon *et al.*, 2016). Research on advance statements shows that Black people with severe mental illness benefit more from advance statements than other groups. The CRIMSON trial showed greater cost-effectiveness of JCPs for Black people compared with white and Asian participants (Thornicroft *et al.*, 2013), arising from reduced inpatient service use. In US research, completing advance statements was a more empowering experience for African Americans than for other ethnic groups (Elbogen *et al.*, 2007), and the demand for these was higher among non-white people (Swanson *et al.*, 2006). In England, stakeholders found advance statements to be important for Black people; however, they may face more barriers in creating them. While a lack of trust in mental health services may create a high demand for advanced statements (Barnett *et al.*, 2019; Byrne *et al.*, 2019; Codjoe *et al.*, 2019; Stephenson *et al.*, 2022), it may also make it harder for service users to discuss negative experiences with services that influence their preferences for care. At the service level, it is important to monitor, on the basis of socioeconomic status and protected characteristics, who is offered to make an advance statement, who takes up this offer, whether the content covers aspects of care related to characteristics such as gender identity and pregnancy in addition to religion and culture, whether this content is followed when the advance statement is consulted, and whether, in general, the advance statement content of disadvantaged socioeconomic groups and those with protected characteristics is followed at the same rate as those without. Otherwise, advance statements may be held largely by well-educated, non-minoritized people and the inequities in care described above may be perpetuated or even widened.

### Healthcare provider knowledge and attitudes

The successful implementation of advance statements relies on healthcare providers’ awareness, understanding and acceptance of these documents. Lack of knowledge or negative attitudes among healthcare professionals can hinder the adoption and use of these measures, limiting their effectiveness (Van Dorn *et al.*, 2006).

### Changing preferences and circumstances

An individual’s mental health preferences and circumstances may change over time. As a result, the contents of advance statements may become outdated or may no longer reflect the patient’s current desires. Regularly revisiting and updating these documents are essential to ensure that they remain relevant and accurately represent an individual’s treatment choices. From another perspective, an advance statement that has not changed over a long period may be regarded as evidence of stable and consistent preferences over time.

### Resource constraints

Implementing advance statements requires adequate resources including time, training and personnel. In resource-constrained settings, healthcare providers may face challenges in dedicating sufficient attention and effort to creating and maintaining documents for all eligible patients. Resource limitations can hinder widespread adoption and effectiveness of these documents.

### Emergency situations and crisis response

During emergencies or rapidly evolving crises, healthcare providers may need to act quickly to ensure the safety and well-being of patients. This urgency might not allow sufficient time for a thorough review of advance statements, potentially leading to decisions that do not align with the individual preferences expressed in the documents. This point applies not only to the advance directives of individuals with mental illnesses but also extends to others. Easy retrieval and access by physicians to these documents should be ensured so that they can be followed even in emergency circumstances, for example, by integrating advance statements into the electronic health record.

### Data privacy and confidentiality

Generally, advance statements contain sensitive personal information about an individual’s mental health history and treatment preferences. Ensuring the confidentiality and security of these documents is crucial for protecting patients’ privacy and preventing potential misuse.

### Family involvement

In some cases, family members may be involved in the development of advance statements or consulted during decision-making. The involvement of family members and trusted individuals in the development of advance statements can help create a supportive network for individuals. Family members who participate in the process gain a better understanding of their loved one’s experiences and preferences, leading to increased empathy and support. While family support can be beneficial, conflicts may arise between the patient’s preferences and the family’s wishes, necessitating careful consideration and communication to respect the patient’s autonomy. It is up to the individual to determine how much authority should be delegated to an appointed trusted person for future decisions in situations where the patient loses decision-making capacity. If a designated family member has not been granted the power to decide on their behalf, their will cannot replace that of the person who created the advance statement.

### Long-term evaluation

Continuous evaluation and research are necessary to assess the long-term impact and effectiveness of advance statements in community treatment of severe mental disorders. Monitoring outcomes and patient experiences can inform improvements and adaptations in the use of these documents.

## Conclusions

Advance statements offer significant benefits and opportunities to patients with severe mental disorders, especially those most at risk for involuntary hospitalization. Empowering individuals to express their treatment preferences and to promote early intervention can enhance autonomy, improve therapeutic relationships, and reduce compulsory admissions. Nevertheless, potential issues, such as legal complexities, communication challenges and the risk of exacerbating rather than mitigating racial inequities in access, experiences and outcomes, as well as resource constraints, must be addressed to ensure the effective and ethical use of these documents. There is currently a notable disparity between the wealth of trial evidence from five countries – the UK (Henderson *et al.*, 2004; Thornicroft *et al.*, 2013), the USA (Swanson *et al.*, 2006), the Netherlands (Ruchlewska *et al.*, 2009), France (Tinland *et al.*, 2022) and Germany (Rixe *et al.*, 2023) – and three systematic reviews (Bone *et al.*, 2019; de Jong Mh *et al.*, 2016; Molyneaux *et al.*, 2019) when compared with routine practice. Therefore, research efforts must shift towards utilizing implementation science to collaboratively design resources for effective implementation, particularly for those who can benefit the most from advance statements (Babatunde *et al.*, 2023). In light of the compelling evidence accumulated over the last two decades, global mental health systems should prioritize the integration of these valuable tools into routine clinical practice.
